# Bromodomain protein BRDT directs ΔNp63 function and super-enhancer activity in a subset of esophageal squamous cell carcinomas

**DOI:** 10.1038/s41418-021-00751-w

**Published:** 2021-03-03

**Authors:** Xin Wang, Ana P. Kutschat, Moyuru Yamada, Evangelos Prokakis, Patricia Böttcher, Koji Tanaka, Yuichiro Doki, Feda H. Hamdan, Steven A. Johnsen

**Affiliations:** 1 Department of General, Visceral and Pediatric Surgery, University Medical Center Göttingen, Göttingen, Germany; 2 Department of Gastroenterological Surgery, Graduate School of Medicine, Osaka University, Osaka, Japan; 3 Gene Regulatory Mechanisms and Molecular Epigenetics Lab, Division of Gastroenterology and Hepatology, Mayo Clinic, Rochester, MN USA

**Keywords:** Cancer genetics, Chromatin, Epigenetics

## Abstract

Esophageal squamous cell carcinoma (ESCC) is the predominant subtype of esophageal cancer with a particularly high prevalence in certain geographical regions and a poor prognosis with a 5-year survival rate of 15–25%. Despite numerous studies characterizing the genetic and transcriptomic landscape of ESCC, there are currently no effective targeted therapies. In this study, we used an unbiased screening approach to uncover novel molecular precision oncology targets for ESCC and identified the bromodomain and extraterminal (BET) family member bromodomain testis-specific protein (BRDT) to be uniquely expressed in a subgroup of ESCC. Experimental studies revealed that BRDT expression promotes migration but is dispensable for cell proliferation. Further mechanistic insight was gained through transcriptome analyses, which revealed that BRDT controls the expression of a subset of ΔNp63 target genes. Epigenome and genome-wide occupancy studies, combined with genome-wide chromatin interaction studies, revealed that BRDT colocalizes and interacts with ΔNp63 to drive a unique transcriptional program and modulate cell phenotype. Our data demonstrate that these genomic regions are enriched for super-enhancers that loop to critical ΔNp63 target genes related to the squamous phenotype such as *KRT14*, *FAT2*, and *PTHLH*. Interestingly, BET proteolysis-targeting chimera, MZ1, reversed the activation of these genes. Importantly, we observed a preferential degradation of BRDT by MZ1 compared with BRD2, BRD3, and BRD4. Taken together, these findings reveal a previously unknown function of BRDT in ESCC and provide a proof-of-concept that BRDT may represent a novel therapeutic target in cancer.

## Introduction

Esophageal cancer is a common malignancy and the 6th leading cause of cancer-related deaths worldwide. The overall 5-year survival rate has remained unchanged for the last few decades, ranging from 15 to 25% [1]. Esophageal squamous cell carcinoma (ESCC), the predominant histological subtype, accounts for 90% of esophageal cancer cases and shows an especially high incidence rate in certain geographical locations such as east Asia [2]. Recently, large-scale genomic and epigenomic studies have revealed the genetic and epigenetic landscape of ESCC and identified recurring mutations or deletions in *TP53*, *CDKN2A*, and *RB1*, and frequent amplifications of *SOX2*, *TP63*, and *FGFR1* [2], making them essential parts of the molecular repertoire defining the “squamous” subtype. Notably, several epigenetic modulators including *CREBBP*, *EP300*, *KMT2C*, and *KMT2D* are also frequently mutated [3]. Although these studies have helped in the molecular characterization of ESCC, they have yet to lead to specific molecular targeted therapies for this particular subtype [4].

Epigenetic regulation is crucial for cells to integrate environmental stimuli and intrinsic regulatory networks and maintain cellular homeostasis. Furthermore, dysregulation of epigenetic regulatory mechanisms contributes to tumorigenesis, tumor progression, and the acquisition of therapeutic resistance [5]. However, unlike genetic alterations, epigenetic alterations are usually reversible, thereby providing an ideal possibility for therapeutic intervention. One family of epigenetic regulators that has emerged as a particularly effective and accessible therapeutic target is the bromodomain and extraterminal (BET) family of epigenetic reader proteins. The BET family comprises BRD2, BRD3, BRD4, and bromodomain testis-specific protein (BRDT), and functions by recognizing acetyl groups on both histones [6] and non-histone proteins [7] via their tandem bromodomains. BRD4, the most well-studied BET protein, binds acetylated histones at promoters and enhancers of its target genes where it promotes productive transcriptional elongation [8]. Notably, BRD4 enrichment is a hallmark of super-enhancers (SEs), long stretches of transcriptionally active chromatin regions displaying a particularly high density of transcription factors and cofactors, which are known to regulate key genes essential for cell fate specification and disease progression [9, 10]. Given the critical role in transcriptional regulation, BET proteins have been shown to play important roles in the development of various diseases including cancer, thus emerging as novel therapeutic targets [11]. Although pan-BET inhibitors are being tested in clinical trials for several different malignancies including lymphoma, breast cancer, and prostate cancer [12], the biological understanding of the different BET family members, especially BRD2, BRD3, and BRDT, in cancer is still very limited.

The concept of precision medicine is based on the assumption that targeted therapies developed against specific cancer-relevant proteins may improve clinical outcome while helping to avoid non-specific adverse effects often caused by standard chemotherapies. Thus, highly specific small-molecule inhibitors are being intensively investigated as the next generation of anticancer therapies [13]. In the case of some malignancies such as breast cancer [14], lung cancer [15], and leukemia [16], such approaches have dramatically increased patient survival rates. However, despite numerous clinical trials, successful targeted therapy options for ESCC remain limited [17]. For example, various tyrosine receptor kinase inhibitors such as inhibitors against *EGFR*, which is often overexpressed in ESCC, have failed to improve survival and displayed varying degrees of side effects [18, 19]. Thus, there is an urgent need to identify novel therapeutic targets with lower toxicity.

In this study, we sought to identify novel therapeutic targets from a comprehensive collection of epigenetic factors, which are tissue-specific and differentially expressed in ESCC. Surprisingly, we identified the testis-specific BET family member BRDT as a putative target that is aberrantly expressed in over 30% of ESCC and important for controlling the migratory potential of ESCC cells. Mechanistically, BRDT colocalizes and cooperates with ΔNp63, a defining factor of the squamous subtype in cancer, to drive the expression of a subset of ΔNp63-dependent genes. The aberrant expression of BRDT rewires and enhances the dependencies of ΔNp63, modulating the expression of SE-associated genes. In conclusion, we show that BRDT is expressed in a subset of ESCC and enhances the ΔNp63-dependent transcriptional program to promote cell migration in ESCC.

## Materials and methods

### Cell culture

Cells were cultured in a humidified incubator supplied with 5% CO_2_ at 37 °C. Roswell Park Memorial Institute medium (RPMI-1640; Invitrogen, CA, USA) with 10% fetal bovine serum (FBS) (Sigma, Munich, Germany) and 1% penicillin/streptomycin (Sigma) was used to culture KYSE70, KYSE180, and TE6 cells. RPMI/F12 medium (Invitrogen) with 5% FBS (Sigma) and 1% penicillin/streptomycin (Sigma) was used to culture KYSE150 cells. Dulbecco’s Modified Eagle medium (Invitrogen) with 10% FBS and 1% penicillin/streptomycin (Sigma) was used to culture HEK293T cells. Knockdown, knockout, overexpression, and proliferation assay are described in Supplementary information. The sequence of siRNA is provided in Supplementary Table S3.

### Migration assay

Cell culture inserts with 8 μm transparent polyester membranes (Corning, Inc, NY, USA) were pre-equilibrated in serum-free medium for 30 min prior to being placed in 24-well companion plates (Corning, Inc). In all, 1 ml of normal medium was placed in the well and 50,000 cells in 500 µL were seeded in the inserts and incubated for 48 hours. The migrated cells were then stained with 1% crystal violet in 20% ethanol for 15 min after removing the remaining non-migrated cells from the inner side of inserts and fixing with methanol for 20 min. Subsequently, inserts were dried, scanned, and quantified with ImageJ.

### Tissue specificity expression analysis

Tissue specificity was evaluated as described [20] with minor modifications. The maximal *p* value of specificity index (pSI) across all tissues was taken to calculate tissue specificity index (TSI). The formula is as follows: TSI = −log_10_(max(pSI)).

### Patient samples, RNA isolation, quantitative real-time PCR (qPCR), RNA-seq library preparation

Thirty-one pairs of fresh tumor and adjacent non-tumor samples of ESCC patients prior to treatment were collected and subjected to snap freezing at Osaka University Hospital, Osaka, Japan. RNA was isolated using QIAzol reagent (Qiagen, Venlo, Netherlands). For RNA-seq library preparation RNA quality was confirmed by electrophoresis, then 500 ng of RNA was used as starting material to prepare RNA-seq libraries using TruSeq RNA library prep kit V2 (Illumina) following the manufacturer’s manual. RNA-seq libraries were quantified using Qubit 2 (Invitrogen) and were subjected to Bioanalyzer 2100 (Agilent) for fragment analysis. The sequencing was performed in the next generation sequencing (NGS) Integrative Genomics Core Unit (NIG) in Göttingen, Germany and the Genome Analysis Core at the Mayo Clinic in Rochester, Minnesota, USA. More details including primer sequences for qPCR (Supplementary Table S4) are provided in supplemental information.

### RNA-seq analysis

Sequencing reads were first subjected to FASTQC (available at https://www.bioinformatics.babraham.ac.uk/projects/fastqc/) for quality control. Reads were then mapped to human genome (hg38) with STAR [21]. After sorting the BAM files using samtools [22], the feature counting was done by HTSeq [23]. The resulting count files were used for differential gene expression analysis with the DESeq2 package [24]. Gene set enrichment analysis (GSEA) was conducted using GSEA program [25]. EnrichR [26] was used to analyze enriched pathways and transcription factors.

### Co-immunoprecipitation (Co-IP)

For endogenous co-IP, KYSE180 cells were treated with 20 nM bortezomib for 12 hours prior to harvesting in co-IP buffer (50 mM Tris-HCl, 1% NP-40, 150 mM NaCl) with the same protease inhibitors used in chromatin immunoprecipitation (ChIP). Cells were then lysed for 10 min on ice and scraped. The cell lysate was sonicated for three cycles of 5 min using a Bioruptor (Diagenode, Liège, Belgium). The sonicated lysate was then centrifuged to collect supernatant which was further split for immunoprecipitation. The pre-clearing process was performed by rotating samples with 60 μL of sepharose beads (50%) at 4 °C for 1 hour. The samples were then centrifuged mildly to collect supernatant. Antibodies were then added to the supernatant and the mix was rotated at 4 °C overnight. The quantity of antibody used in this study is provided in Supplementary Table S5. 50 μL of protein G-coupled sepharose beads (50%) (GE healthcare, Chicago, IL, USA) was then added to samples and the samples were incubated at 4 °C for 2 hours to capture the immune complex. Subsequently, samples were centrifuged and washed three times with co-IP buffer. Finally, the collected beads were eluted by adding 25 μL of Laemmli buffer and subjected to western blot for analyzing protein interactions.

For exogenous co-IP, transfected HEK293T cells were processed using the same protocol as the endogenous co-IP. However, the IP step was done using GFP-Trap Agarose (ChromoTek, Germany) and anti-FLAG M2 Affinity Gel (Sigma, St. Louis, MO, USA) for GFP and FLAG IP, respectively.

### ChIP and ChIP-seq library preparation

ChIP was done as previously described [27, 28] with minor changes. Cells were washed with phosphate-buffered saline (PBS) and cross-linked using 1% formaldehyde in PBS for 10 min. After quenching the formaldehyde with 1.25 mM glycine, fixed cells were washed twice with ice-cold PBS. Cells were then lysed using nuclear preparation buffer supplemented with protease inhibitors to isolate nuclei. After brief centrifugation, nuclei were resuspended in lysis buffer containing protease inhibitors. Subsequently, samples were sonicated for 12 cycles using a Bioruptor (Diagenode). Chromatin extracts were centrifuged and pre-cleared with sepharose 4B (GE Healthcare) beads for 1 hour. After centrigugation, the antibody was then added to the pre-cleared chromatin and incubated overnight with rotation. Details about the antibodies used in this study are provided in Supplemental Table S5. Sepharose beads coupled with Protein A or Protein G (GE Healthcare) were then added to the reaction and incubated for 2 hours to capture immunocomplexes. Samples were then washed with lysis buffer, wash buffer, and Tris-EDTA (TE) buffer. De-crosslinking was done by incubating with 20 µg proteinase K overnight at 55 C. The DNA was extracted using phenol/choloroform/isoamyl alcohol (25:24:1) and precipitated using ethanol.

Chromatin immunoprecipitation-sequencing (ChIP-seq) library preparation was done using KAPA Hyper Prep Kit (Roche, Basel, Switzerland). ChIP DNA was quantified with Qubit (Invitrogen) and libraries were prepared according to the manufacturer’s instructions. The library concentration and fragment size were determined by Qubit and Bioanalyzer, respectively. Sequencing was performed in the NIG, Göttingen, Germany.

### ChIP-seq bioinformatic analysis

Sequencing reads were mapped to the human genome (hg19) using bowtie [29]. The resulting bam files were sorted and indexed using samtools [22]. Deeptools [30] was used to convert bam files to signal tracks. The bigwig file of BRDT was smoothened by averaging five consecutive bins. MACS2 [31] was utilized to identify peaks and motif analysis was run using the HOMER suite [32]. Notably, BRDT peaks were called using the following parameters: --broad --broad-cutoff 0.1 --llocal 50,000 due to relatively low signal/background ratio. The identification of SEs was carried out using ROSE [9, 33]. ChromHMM [34] was used to analyze the histone modification pattern across the genome.

### H3K27ac HiChIP

HiChIP was done as previously described [35] with some changes. Cells were washed twice with PBS and cross-linked using 1% formaldehyde in PBS for 10 min, which was quenched by incubating with 1.25 mM glycine solution for 5 min. The cross-linked cells were washed twice with ice-cold PBS and lysed with HiC lysis buffer. The nuclei were collected and resuspended in 0.5% sodium dodecyl sulfate, which was then quenched by adding 10% Triton X-100. The digestion was carried out by incubating with 200 U of MboI, DpnII, and HinfI (NEB, Ipswich, MA, USA) at 37 °C for 2 hours. After heat inactivation of restriction enzymes at 62 °C for 10 min, the overhangs of digested chromatin were filled by adding dCTP, dGTP, dTTP, and biotin-labeled dATP (Jena biosciences, Jena, Germany) and DNA Polymerase I large (Klenow) fragment (NEB). After the biotin incorporation, proximity ligation was performed using T4 DNA Ligase (NEB). The samples were then resuspended in lysis buffer supplemented with protease inhibitors. To further solubilize chromatin, four cycles of sonication were applied. The size distribution of DNA fragments was verified by agarose gel electrophoresis before pre-clearing the chromatin with 50% sepharose 4B (GE Healthcare) slurry in lysis buffer. H3K27ac-associated chromatin was captured by adding 6 μg of H3K27ac antibody (Diagenode). Protein A-sepharose (GE Healthcare) beads were added to capture the immunocomplex. The beads were subsequently washed with lysis buffer, wash buffer, lysis buffer, and TE buffer, and subjected to DNA extraction with phenol/chloroform/isoamyl alcohol (25:24:1) as described for ChIP. Right-sided size selection using KAPApure beads (Roche) was performed according to the manufacturer’s guidelines to exclude large DNA fragments prior to library preparation. Libraries were prepared following the KAPA Hyper Prep manual. Streptavidin T-1 beads (Invitrogen) were washed with Tween wash buffer and resuspended in biotin binding buffer to capture biotin-labeled DNA. Library amplification was carried out according to the KAPA Hyper Prep manual. The fragment distribution of HiChIP libraries was determined using a Bioanalyzer (Agilent, Santa Clara, CA, USA). The libraries were then sequenced by the Genome Analysis Core at the Mayo Clinic, Rochester, MN.

### HiChIP bioinformatic analysis

HiChIP data were analyzed using the HiC-Pro pipeline [36], which includes read alignment, HiC read filtering, quality checks, and contact matrix building. FitHiChIP [37] was utilized to identify active p63-associated loops. The “Peak-To-All” mode was used and the resulting loops were further processed to exclude those of which either end is not marked by H3K27ac. Subsequently, the result was converted to bedpe format for downstream visualization and enhancer-gene association. The enhancer-gene association was done using in-house scripts and the link to the source code can be found in the section of “data availability”.

### Statistical analyses

Data are presented as mean±SD. Statistical methods, number of replicates, and significance are indicated in each experiment.

## Results

### Unbiased screening identified BRDT expression in a subset of ESCC

**Fig. 1:**
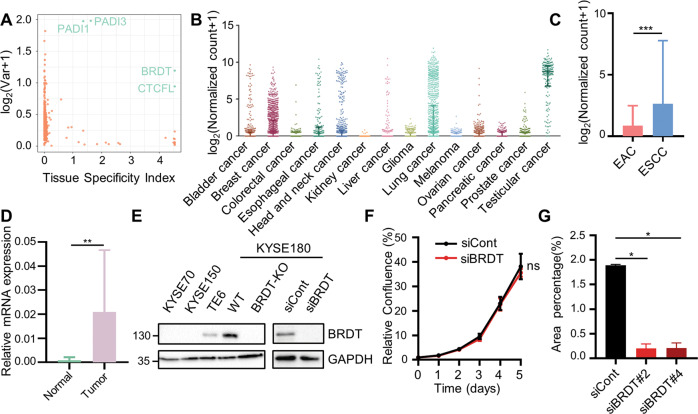
Unbiased screening identifies BRDT as a potential therapeutic target for precision medicine in ESCC. **A** Scatter plot showing tissue specificity in normal tissues (*y* axis) and expression variance (x axis) of epigenetic factors in ESCC. **B** Expression of *BRDT* in different cancer entities. **C** Box plot showing 10–90 percentile of the expression of *BRDT* in different histological subtypes. Unpaired *t* test was used. **D** Quantitative real-time PCR analysis of *BRDT* expression in tumor and adjacent normal tissues presented with box plot showing 10–90 percentile. Samples of 31 patients were evaluated. Paired *t* test was used. *ACTB* was used to normalize gene expression. **E** Western blot analysis of BRDT in various ESCC cell lines and siRNA-mediated knockdown of BRDT and CRISPR/Cas9-mediated knockout of *BRDT* in KYSE180 cells. **F** Growth kinetics analysis of control (siCont) and BRDT knockdown (siBRDT) in KYSE180. Data are represented as mean±SD, *n* = 4. Paired *t* test was used. **G** Quantification of migrated cells upon BRDT knockdown with different siRNAs in KYSE180. Data are represented as mean±SD, *n* = 2. Unpaired *t* test was used. ****P ≤ 0.0001, ***P ≤ 0.005, **P ≤ 0.01, *P ≤ 0.05, ns: not significant.

Owing to their reversible nature and potential targetability, epigenetic modulators represent ideal candidates for anticancer therapy. In order to uncover potential targets for ESCC treatment that elicit minimal side effects, we sought to identify targetable epigenetic regulators, which are tissue-specific and differentially expressed in ESCC. In order to achieve this, we exploited publicly available expression data [4, 20] and identified four genes (*PADI1*, *PADI3*, *BRDT*, *CTCFL*), which displayed high levels of tissue specificity and differential expression in ESCC (Fig. 1A, Supplementary Table S1). Given the potential targetability of BRDT by small-molecule BET inhibitors, we further investigated this testis-specific member of the BET family of proteins. To date, most studies examined BRDT function in male germ cells [38–42]. However, we found *BRDT* to be aberrantly expressed in >30% of ESCC [43] (Supplementary Table S2). To more generally explore the expression pattern of BRDT in cancer, we leveraged data from The Cancer Genome Atlas (TCGA) consortium and observed that *BRDT* is significantly expressed in several malignancies in addition to esophageal cancer and testicular cancer, including breast, lung, and head and neck cancers (Fig. 1B). We next investigated whether BRDT was preferentially expressed in a certain histological subtype of esophageal cancer and found *BRDT* to be preferentially expressed in ESCC (Fig. 1C, Supplementary Table S2). Notably, consistent with the TCGA data, we were able to confirm that *BRDT* is expressed in an independent cohort of ESCC compared with adjacent normal tissue (Fig. 1D). Moreover, using data from the Cancer Cell Line Encyclopedia [44] we identified two BRDT-positive (KYSE180 and TE6) and two BRDT-negative ESCC cell lines (KYSE70 and KYSE150) and confirmed BRDT expression in KYSE180 and TE6 cells (Fig. 1E). In order to examine potential tumorigenic functions of BRDT, we utilized CRISPR/Cas9-mediated genome editing and siRNA-mediated knockdown to efficiently deplete BRDT protein levels (Fig. 1E). Although genetic deletion or siRNA-mediated silencing of BRDT did not appreciably affect cell proliferation, migration potential was largely abolished, suggesting a role of BRDT in controlling cell migration (Fig. 1F, G, Supplementary Fig. S1A-D). Together, these results indicate that BRDT is aberrantly expressed in a subset of ESCC and may function to promote cell migration.

### BRDT regulates gene expression programs related to cell migration in ESCC

**Fig. 2:**
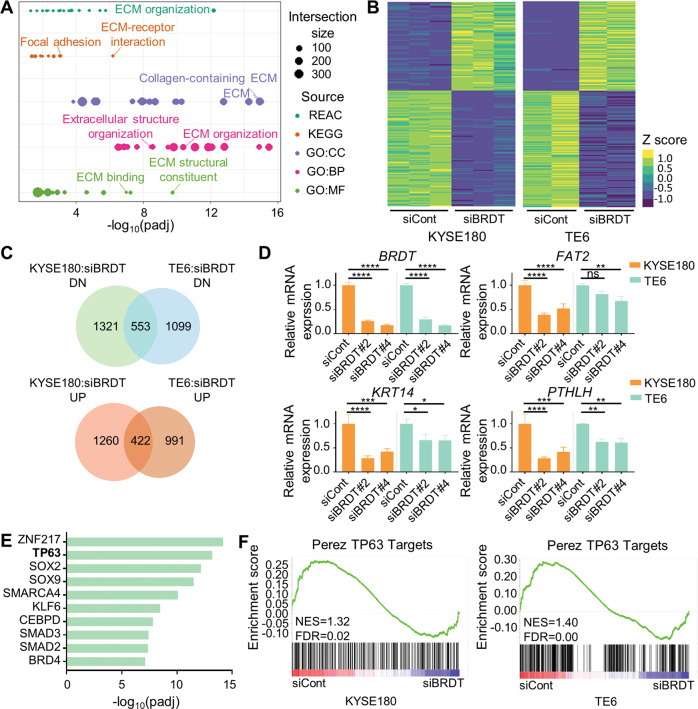
Transcriptomic profiling reveals the role of BRDT in cell migration. **A** Pathway analysis of downregulated genes upon BRDT knockdown in KYSE180. Dots on the graph indicate enriched pathways. Intersection size: the overlap between the query list and the annotation terms from different sources. *REAC* reactome, *KEGG* Kyoto Encyclopedia of Genes and Genomes, *GO:CC* gene ontology: cellular component, *BP* biological process, *MF* molecular function. **B** Heatmaps showing the robustness of the RNA-seq data in KYSE180 (left) and TE6 (right). The commonly regulated (*p*adj < 0.05 and log2FC ≤ −0.5 or log2FC ≥ 0.5) genes of KYSE180 and TE6 are plotted. **C** Venn diagrams showing the overlap of significantly regulated (*p*adj<0.05 and log2FC ≤ −0.5 or log2FC ≥ 0.5) genes between KYSE180 and TE6. **D** Quantitative real-time PCR validation of downregulated genes upon BRDT knockdown in KYSE180 and TE6. *GAPDH* was used to normalize gene expression. Data are represented as mean±SD, *n* = 3. Unpaired one-way ANOVA test followed by Dunnett’s test was used. ****P ≤ 0.0001, ***P ≤ 0.005, **P ≤ 0.01, *P ≤ 0.05, ns: not significant. **E** ChIP enrichment analysis (ChEA) showing enriched factors of commonly downregulated genes between KYSE180 and TE6. **F** GSEA showing TP63 target genes are regulated by BRDT in KYSE180 and TE6.

In order to gain mechanistic insight into the role of BRDT in ESCC, we performed mRNA-seq upon depletion of BRDT in KYSE180 cells. As BET proteins generally function as transcriptional activators, we performed pathway enrichment analysis on genes downregulated following BRDT depletion. This approach identified extracellular matrix (ECM) organization-related pathways (Fig. 2A), processes critical for cell migration [45, 46], as being key downstream targets of BRDT. RNA-seq analysis of a second BRDT-positive ESCC cell line (TE6) revealed a significant overlap between the regulated genes in the two different cell lines (Fig. 2B, C) and could be experimentally validated in both cell systems (Fig. 2D).

As BET proteins bind to acetylated lysines and do not possess intrinsic sequence-specific DNA-binding capacity, we sought to identify specific transcription factors associated with BRDT-dependent transcriptional regulation. Strikingly, when examining transcription factors enriched on genes downregulated upon BRDT depletion, we identified TP63 as a top candidate (Fig. 2E). This finding is consistent with the TP63 isoform ΔNp63 being a key regulator of the squamous-specific transcriptional program [2, 28, 47, 48]. Consistently, GSEA also identified TP63-related gene signatures as being downregulated in BRDT-depleted KYSE180 and TE6 cells (Fig. 2F). These results uncover BRDT as a novel regulator of cell migration-related and ΔNp63-dependent gene programs in ESCC.

### BRDT occupies epigenetically active genomic regulatory regions

**Fig. 3:**
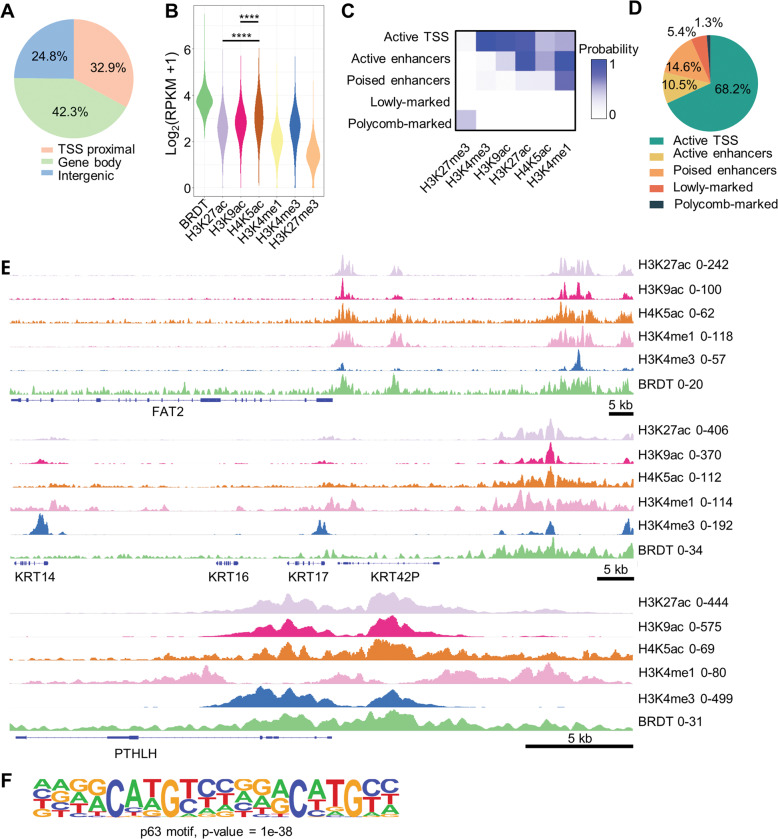
ChIP-seq uncovers the genomic occupancy of BRDT in KYSE180. **A** Analysis of genomic occupancy of BRDT. **B** Violin plot showing the signal strength of BRDT on various histone modification-bound regions. Unpaired one-way ANOVA test followed by Dunnett’s test was used. ****P ≤ 0.0001, ***P ≤ 0.005, **P ≤ 0.01, *P ≤ 0.05, ns: not significant. **C** ChromHMM analysis identifying different chromatin states based on histone modification patterns. **D** Distribution of BRDT over different chromatin states. **E** ChIP-seq tracks of BRDT and other histone marks at *FAT2*, *KRT14*, and *PTHLH* loci. **F** Motif analysis of BRDT bound regions identifying p63 motif.

Although BRDT occupancy was previously examined in germ cells [41], its role in gene regulation and genome occupancy has not been investigated in tumor cells to date. In order to dissect the function of BRDT in controlling gene expression in ESCC, we performed ChIP-seq analyses of BRDT in KYSE180 cells. These results revealed that BRDT is localized both to promoter-proximal and distal enhancer regions (Fig. 3A). As BET proteins have a high-affinity towards diacetylated histone 4 (H4) tails [49], we also performed epigenome mapping studies for several histone modifications. Specifically, we examined the occupancy of H4K5ac, H3K9ac, H3K27ac, H3K4me1, H3K4me3, and H3K27me3 in KYSE180 cells. We found that BRDT preferentially colocalizes with active histone marks such as H3K27ac, H3K9ac, H4K5ac, H3K4me1, and H3K4me3, further supporting a positive role for BRDT in regulating gene expression. Moreover, consistent with biophysical studies showing that murine BRDT has a binding preference for acetylated H4 [50], we observed a higher concordance of BRDT occupancy with H4K5ac compared with either H3K27ac or H3K9ac (Fig. 3B). To gain more insight into the epigenomic context of BRDT occupancy, we classified the genome into different chromatin states based on the investigated histone marks (Fig. 3C) and examined the overlap of BRDT-enriched regions with each defined state. This revealed that BRDT is mainly localized to active transcription start sites (TSSs) and enhancers (Fig. 3D, E), providing further support that BRDT is a positive transcriptional regulator.

In order to identify potential transcription factors directing BRDT activity in ESCC, we performed motif analyses on BRDT-enriched genomic regions. Consistent with the results of our transcriptome data, consensus motifs bound by TP63 were highly enriched in BRDT-occupied regions (Fig. 3F). Together these results illustrate that BRDT preferentially binds to active TSS and enhancers and support its potential role in directing ΔNp63 activity in ESCC.

### BRDT colocalizes with the squamous transcription factor ΔNp63

**Fig. 4:**
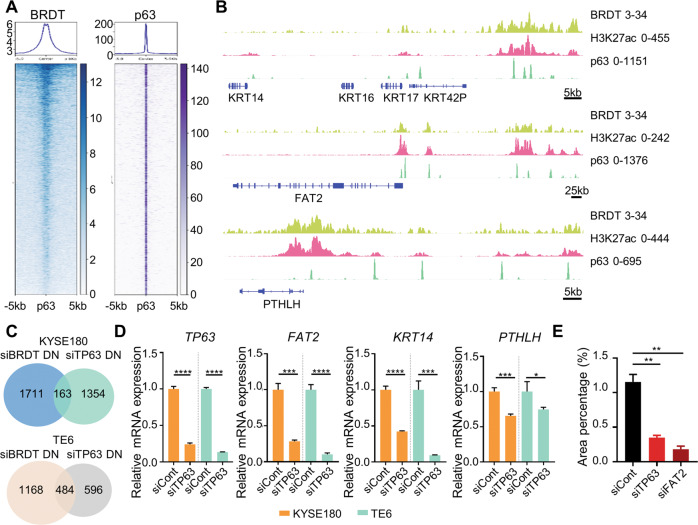
BRDT colocalizes with the squamous transcription factor ΔNp63. **A** Heatmaps showing the co-occupancy of BRDT and p63. Plots are centered on summits of active p63-bound (co-occupied by p63 and H3K27ac) regions. **B** ChIP-seq tracks showing the colocalization of BRDT and p63 at *FAT2*, *KRT14*, and *PTHLH* loci. **C** Venn diagrams showing the overlap between BRDT- and p63-targets in KYSE180 (left) and TE6 (right). **D** Quantitative real-time PCR analysis of *FAT2*, *KRT14*, and *PTHLH* upon knockdown of p63 in KYSE180. *GAPDH* was used to normalize gene expression. Data are represented as mean±SD, *n* = 3. Unpaired *t* test was used. **E** Quantitation of migrated cells upon knockdown of p63 and FAT2 in KYSE180. Data are represented as mean±SD, *n* = 2. Unpaired one-way ANOVA test followed by Dunnett’s test was used. ****P ≤ 0.0001, ***P ≤ 0.005, **P ≤ 0.01, *P ≤ 0.05, ns: not significant.

Given our findings that BRDT is required for the expression of a p63-controlled transcription program and enrichment of a p63-binding motif in BRDT-occupied genomic regions, we hypothesized that BRDT and ΔNp63 may functionally interact with one another. To address this, we performed ChIP-seq analysis for ΔNp63 in KYSE180 and examined its colocalization with BRDT. Strikingly, BRDT co-occupied many active ΔNp63-bound regions (i.e., ΔNp63-bound regions marked by H3K27ac), supporting a functional interplay between BRDT and ΔNp63 (Fig. 4A). Individual examples of genes co-occupied by BRDT and ΔNp63 included *KRT14*, *FAT2*, and *PTHLH*, whose expression is ΔNp63-dependent and tightly associated with a squamous gene expression program (Fig. 4B). Based on the co-occupancy of ΔNp63 and BRDT we hypothesized that the two proteins may form a complex to execute transcriptional regulatory roles. In order to test this, we performed endogenous co-IP in KYSE180 as well as exogenous co-IP in HEK293T cells. Indeed, immunoprecipitation of BRDT resulted in a co-IP of ΔNp63 (Supplementary Fig. S2A, left panel) and vice versa (Supplementary Fig. S2A, right panel).

Given our initial finding that BRDT was required for the expression of published p63-dependent genes and evidence of cooperation across the genome of ESCC cells, we next sought to validate the cooperative function of BRDT and ΔNp63 in ESCC by examining the effects of depleting ΔNp63 on transcription. Consistent with the notion that BRDT has a central role in regulating ΔNp63 activity, we found an overlap between BRDT- and ΔNp63-dependent genes in both KYSE180 and TE6 (Fig. 4C). Exemplary, three genes co-occupied by BRDT and ΔNp63 (*FAT2*, *KRT14*, and *PTHLH*), could be confirmed to be downregulated upon depletion of either BRDT (Fig. 2D) or ΔNp63 (Fig. 4D). Moreover, KRT14 protein levels were also decreased following depletion of either BRDT or ΔNp63 (Supplementary Fig. S2B). Given our observation that BRDT was required for cell migration, we hypothesized that depletion of either the responsible transcription factor providing sequence specificity to BRDT activity (ΔNp63) or a downstream ΔNp63/BRDT target previously shown to control cell migration in human squamous carcinoma cells (*FAT2*) [51], may phenocopy the effects of BRDT depletion on cell migration. Remarkably, we observed that the depletion of either ΔNp63 or FAT2 significantly decreased cell migration (Fig. 4E, Supplementary Fig. S2C). Taken together, these results suggest that ΔNp63 is associated with and functionally cooperates with BRDT to transcriptionally activate genes essential for cell migration.

### BRDT directs and rewires ΔNp63-dependent transcription in ESCC

**Fig. 5:**
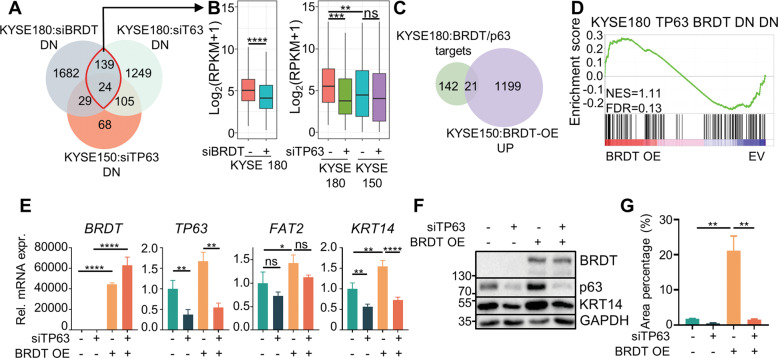
BRDT directs and rewires ΔNp63 programs. **A** Venn diagram showing the overlap between the BRDT/p63-targets in KYSE180 and p63-targets in KYSE150. Red circle denotes BRDT/p63-targets. **B** Boxplots showing the expression of BRDT/p63-targets in different conditions in KYSE180 and KYSE150. Paired *t* test was used for the left panel and paired one-way ANOVA test followed by Tukey’s test was used for right panel. **C** Venn diagram showing the overlap between the BRDT/p63-targets of KYSE180 and BRDT-activated genes of KYSE150. **D** GSEA showing that BRDT/p63-targets are enriched in KYSE150 overexpressing BRDT. *EV* empty vector. **E** Quantitative real-time PCR analysis of *FAT2* and *KRT14* upon overexpression of BRDT and knockdown of p63 in KYSE150. *GAPDH* was used to normalize gene expression. Data are represented as mean±SD, *n* = 3. Unpaired one-way ANOVA test followed by Tukey’s test was used. **F** Western blot analysis of KRT14 upon overexpression of BRDT and/or knockdown of p63 in KYSE150. **G** Quantitation of migrated cells upon overexpression of BRDT and/or knockdown of p63 in KYSE150. Data are represented as mean±SD, *n* = 2. Unpaired one-way ANOVA test followed by Tukey’s test was used. ****P ≤ 0.0001, ***P ≤ 0.005, **P ≤ 0.01, *P ≤ 0.05, ns: not significant.

The expression of ΔNp63 is a common feature among squamous cell carcinomas, including ESCC. Thus, we were interested in determining the specificity of BRDT in controlling ΔNp63-dependent transcription and the impact of BRDT on the ΔNp63-dependent program. Therefore, we depleted ΔNp63 in KYSE150, which lack endogenous BRDT expression, and compared this dataset with genes downregulated following BRDT and ΔNp63 depletion in KYSE180 cells. Strikingly, we found that BRDT/ΔNp63-dependent genes displayed limited overlap with ΔNp63 targets from KYSE150 (Fig. 5A), indicating that BRDT may function to reprogram ΔNp63 dependencies in ESCC. We further compared the expression level of BRDT/ΔNp63 targets and found that these genes are more highly expressed in KYSE180 compared with KYSE150 and were not regulated by ΔNp63 in KYSE150 (Fig. 5B), further supporting that BRDT specifically reprograms the ΔNp63-dependent transcriptional program. To further investigate the ability of BRDT to reprogram ΔNp63 dependencies, we performed RNA-seq in KYSE150 cells overexpressing BRDT. In accordance with our hypothesis, a subset of BRDT/ΔNp63 targets was upregulated upon overexpression of BRDT in KYSE150 cells (Fig. 5C). Consistently, many BRDT/ΔNp63 target genes were enriched in cells overexpressing BRDT, suggesting that overexpressing BRDT in a BRDT-negative cell line is sufficient to partially reprogram the ΔNp63-dependent transcriptional program (Fig. 5D). To further confirm the importance of ΔNp63 in directing BRDT function, we depleted ΔNp63 in either control KYSE150 cells or cells overexpressing BRDT and examined ΔNp63/BRDT target gene expression. Consistent with our model in which ΔNp63 directs BRDT to target genes, we observed that depletion of ΔNp63 precludes the ability of BRDT to activate the expression of either *KRT14* or *FAT2* (Fig. 5E). Consistent with changes observed at the mRNA level, we also found that KRT14 protein levels are also regulated in the same manner (Fig. 5F). Importantly, consistent with the functional importance of BRDT in controlling tumor cell migration, BRDT overexpression in KYSE150 increased cell migration and this effect was blocked by depleting ΔNp63 (Fig. 5G, Supplementary Fig. S3). Collectively, our resutls show that BRDT rewires the ΔNp63-dependent transcriptional program in ESCC.

### BRDT controls ΔNp63-dependent SEs

**Fig. 6:**
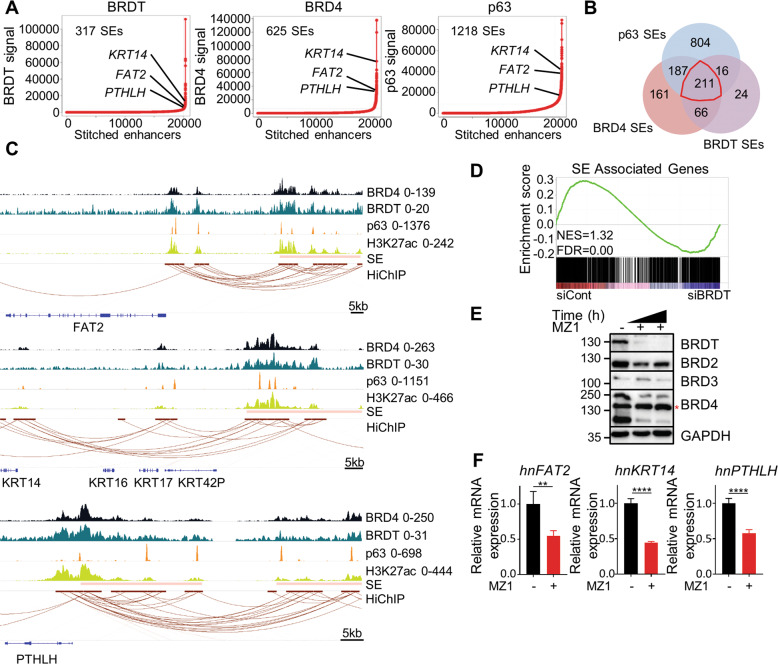
BRDT controls ΔNp63-dependent super-enhancers. **A** Super-enhancer calling using BRDT, BRD4, and p63, respectively. **B** Venn diagram showing the overlap among BRDT-, BRD4-, and p63-super-enhancers. **C** Tracks showing BRDT, BRD4, p63, H3K27ac, super-enhancers (SE) and H3K27ac HiChIP interactions at *FAT2*, *KRT14*, and *PTHLH* loci. **D** GSEA showing genes associated with super-enhancers are enriched in control group in KYSE180. **E** Western blot analysis of BET proteins upon 4 h and 8 h of 1 µM MZ1 treatment in KYSE180. *: non-specific band. **F** Quantitative real-time PCR analysis of heterogeneous nuclear RNA of *FAT2*, *KRT14*, and *PTHLH* upon 8 h of 1 µM MZ1 treatment in KYSE180. *GAPDH* was used to normalize gene expression. Data are represented as mean±SD, *n* = 4. Unpaired *t* test was used. ****P ≤ 0.0001, ***P ≤ 0.005, **P ≤ 0.01, *P ≤ 0.05, ns: not significant.

We and others previously demonstrated that ΔNp63 plays a central role in determining tumor cell identity by controlling SEs to modulate target gene expression [9, 28, 52, 53]. Given our findings that BRDT colocalized with ΔNp63 on several genes such as *FAT2* and *PTHLH*, which we previously demonstrated as being associated with ΔNp63-dependent SEs in pancreatic cancer [28], we investigated whether BRDT, like BRD4, may be a defining feature of SEs in a subset of ESCC. For this, we compared the ability of BRD4, BRDT, or ΔNp63 occupancy to identify SEs on stitched H3K27ac peaks [9, 33] (Fig. 6A). Strikingly, we found that >60% of the BRDT-occupied SEs overlap with those identified by either BRD4 or ΔNp63 occupancy (Fig. 6B).

Recent studies have revealed that SEs direct specific transcriptional programs via chromatin loops with the promoters of important target genes [9, 54, 55]. In order to accurately identify genes associated with BRDT SEs, we utilized HiChIP [35] to capture chromatin interactions associated with active (H3K27ac occupied) chromatin regions in KYSE180. Interestingly, the identified BRDT SEs are associated with key BRDT/ΔNp63-dependent subtype-specific and migration-associated genes such as *FAT2*, *KRT14*, and *PTHLH* (Fig. 6C). We further exploited this HiChIP data to identify genes associated with SEs and found these genes to be enriched in control KYSE180 cells compared with the BRDT-depleted group (Fig. 6D), highlighting the role of BRDT in directing SE function.

BET proteins have provided an important paradigm as therapeutic epigenetic targets in cancer [9]. Thus, given its amenability to BET inhibitor treatment [40, 41], BRDT may therefore represent an attractive novel target for precision medicine in ESCC. In particular, proteolysis-targeting chimeric (PROTAC) molecules represent novel candidates for anticancer therapy [56]. Notably, the VHL-dependent PROTAC MZ1 was reported to display specificity towards BRD4 in comparison with BRD2 and BRD3 [57]. However, to what degree it affects BRDT is currently unknown. Interestingly, our results demonstrate that BRDT was completely degraded after a brief treatment with 1 µM MZ1, whereas BRD4 expression was greatly, but not completely decreased, and BRD2 and BRD3 protein levels were comparatively unaffected (Fig. 6E). Based on these findings we tested whether MZ1 treatment can also downregulate the expression of BRDT/ΔNp63 targets. Consistent with the effects of MZ1 on BRDT protein levels, MZ1 treatment resulted in decreased expression of nascent (heterogeneous nuclear) RNA (hnRNA) of *KRT14*, *FAT2*, and *PTHLH* following MZ1 treatment (Fig. 6F), resembling the effects observed following the knockdown of BRDT or ΔNp63. Together, these results show that BRDT occupies a subset of ΔNp63-dependent SEs to modulate squamous-specific gene expression in a subset of ESCC.

## Discussion

Current therapeutic approaches for the treatment of ESCC display highly heterogeneous efficacies and frequently elicit undesirable side effects. The identification of therapeutic targets with high tissue specificity would offer a unique approach to cancer therapy with low-toxicity and decreased side effects. In this work, we sought to ascertain such targets by identifying variably expressed tissue-specific epigenetic factors aberrantly expressed in ESCC. Utilizing an unbiased approach, we identified *BRDT*, the testis-specific member of the BET family proteins, as one of the most variably expressed tissue-specific genes in ESCC. Although depletion of BRDT did not impair cell proliferation, it did result in attenuated cell migration and downregulation of related pathways. Mechanistically, we demonstrate for the first time that BRDT occupancy is associated with the activity of a select set of cancer subtype-specific genes. Integrative analyses of the transcriptomic and genomic occupancy data led us to the finding that BRDT acts at a subset of SEs to maintain the expression of cell lineage-specific genes.

BRDT has been reported to function as a master regulator during spermatogenesis by inducing massive chromatin reorganization [38]. Interestingly, BRDT was also reported to be ectopically expressed in cancer two decades ago [58], but its function in tumorigenesis has remained elusive until now. Two recent studies published during the revision of this work showed a role of BRDT in regulating tumor growth both in vitro and in vivo [59, 60], highlighting the clinical potential of BRDT. However, neither study revealed the underlying mechanism by which BRDT functions in cancer. In this study, we show for the first time that BRDT can regulate transcription by promoting ΔNp63 function at certain SEs. This regulatory mechanism is similar to what we have previously shown for BRD4, the closest paralog to BRDT, which localizes to lineage-specific enhancers to regulate genes that are crucial for lineage specification [27] and pancreatic cancer subtype [28]. Consistently, we report that BRDT localizes to a select subset of ΔNp63-bound SEs, serving to maintain the expression of the associated squamous-specific genes. Our data suggest that BRDT forms a complex with ΔNp63. However, whether this association is direct or indirect remains unknown. It is likely that other factors influencing squamous cell carcinoma phenotype such as SOX2, which also appears as one of the top BRDT-associated factors, may be involved. Thus, it is possible that BRDT, SOX2, and ΔNp63 may cooperate at certain enhancer regions to promote squamous-specific transcription and maintain the squamous phenotype. Although the precise function of BRDT at enhancers remains to be determined, like BRD4, BRDT possesses an extended carboxyl terminus that can interact with the Positive Transcription Elongation Factor-b (P-TEFb) [41]. Thus, it is likely that BRDT may function to control enhancer activity via regulation of promoter-proximal pausing and/or enhancer RNA synthesis, both of which are primarily controlled by the P-TEFb subunit CDK9 in conjunction with BET proteins [8, 61].

A number of studies have reported an antitumor activity of BET inhibition in preclinical models [62–65], thus leading to numerous ongoing early phase clinical trials of BET inhibitors in various cancers. A phase I clinical study has already shown that BET inhibitors can have clinical efficacy in different cancers [66]. This highlights the potential of BET inhibition as a therapeutic approach for cancer treatment. However, BET inhibitors elicit a number of side effects related to their physiological roles in hematopoietic cell lineage specification [67]. Recently, new inhibitors developed specifically against the second bromodomain of BRD4 show much lower toxicity, but also a more-limited spectrum of malignant indications [68, 69]. Nevertheless, these studies showed the feasibility of developing specific inhibitors for individual bromodomains of BET proteins, thereby suggesting that specific targeting of BRDT may be feasible. Another potential therapeutic approach is through the utilization of BET isoform-specific PROTACs. In general, BET degraders confer a more profound effect on BET-mediated transcriptional modulation, thus leading to a stronger antitumor activity [70, 71]. One notable example is MZ1, a PROTAC BET degrader, which was previously shown to preferentially degrade BRD4 over BRD2 and BRD3 [57]. Interestingly, our results demonstrate that MZ1 efficiently induces BRDT degradation to an extent even greater than BRD4 and efficiently downregulates BRDT-dependent transcriptional targets. Thus, specific inhibition or degradation of BRDT represents a unique opportunity with a strong potential for clinical application in ESCC.

In our study, BRDT was specifically required for cell migration, but dispensable for cell proliferation. Therefore, despite the potential utility of small-molecule inhibitors or PROTACs in blocking BET protein function, it is currently unclear whether the inhibition or depletion of BRDT activity would be sufficient to impede ESCC tumor growth. Thus, although such inhibitors would be predicted to limit tumor metastasis, a different approach would likely be required to more efficiently impede tumor growth. One potential approach may be the conjugation of anti-neoplastic substances such as chemotherapeutic agents or radionuclides to a BRDT-specific ligand. Such an approach would not only enable highly specific targeting of BRDT-expressing tumor cells but could also facilitate non-invasive imaging of tumors. Similar proof-of-principle molecules have been developed for hormone-dependent cancers such as breast and prostate cancer by utilizing specific conjugates of estrogen and androgen receptor ligands, respectively [72, 73]. Importantly, given the unique tissue specificity of BRDT expression during spermatogenesis, it is anticipated that any side effects due to its specific targeting will both be minimal and reversible.

Taken together, our unbiased screening of epigenetic factors led us to the identification of BRDT as an unexpected and novel potential therapeutic target in ESCC. Future studies will be needed to identify and refine small-molecule probes targeting BRDT and test their utility in preclinical models and early clinical trials.

## Supplementary information

Supplementary Information

Supplementary Table S1

Supplementary Table S2

## Data Availability

The NGS data generated during the current study are available in the Gene Expression Omnibus repository under accession number: GSE155187. The script utilized for enhancer-promoter interaction identification can be accessed at https://github.com/BoxWong/SE-association/blob/master/SE_asso_HiChIP.py.

## References

[1] Siegel RL, Miller KD, Jemal A (2019). Cancer statistics, 2019. CA Cancer J Clin.

[2] Smyth EC, Lagergren J, Fitzgerald RC, Lordick F, Shah MA, Lagergren P (2017). Oesophageal cancer. Nat Rev Dis Prim.

[3] Gao Y-BB, Chen Z-LL, Li J-GG, Hu X-DDa, Shi X-JJ, Sun Z-MM (2014). Genetic landscape of esophageal squamous cell carcinoma. Nat Genet.

[4] Lin DC, Wang MR, Koeffler HP (2018). Genomic and epigenomic aberrations in esophageal squamous cell carcinoma and implications for patients. Gastroenterology.

[5] Morel D, Jeffery D, Aspeslagh S, Almouzni G, Postel-Vinay S (2020). Combining epigenetic drugs with other therapies for solid tumours—past lessons and future promise. Nat Rev Clin Oncol.

[6] Belkina AC, Denis GV (2012). BET domain co-regulators in obesity, inflammation and cancer. Nat Rev Cancer.

[7] Shi J, Wang Y, Zeng L, Wu Y, Deng J, Zhang Q (2014). Disrupting the interaction of BRD4 with diacetylated twist suppresses tumorigenesis in basal-like breast cancer. Cancer Cell.

[8] Moon KJ, Mochizuki K, Zhou M, Jeong H-SS, Brady JN, Ozato K (2005). The bromodomain protein Brd4 is a positive regulatory component of P-TEFb and stimulates RNA polymerase II-dependent transcription. Mol Cell.

[9] Lovén J, Hoke HA, Lin CY, Lau A, Orlando DA, Vakoc CR (2013). Selective inhibition of tumor oncogenes by disruption of super-enhancers. Cell.

[10] Sabari BR, Dall’Agnese A, Boija A, Klein IA, Coffey EL, Shrinivas K (2018). Coactivator condensation at super-enhancers links phase separation and gene control. Science.

[11] Dawson MA, Kouzarides T, Huntly BJP (2012). Targeting epigenetic readers in cancer. N. Engl J Med.

[12] Stathis A, Bertoni F (2018). BET proteins as targets for anticancer treatment. Cancer Discov.

[13] Sawyers C (2004). Targeted cancer therapy. Nature.

[14] Goutsouliak K, Veeraraghavan J, Sethunath V, De Angelis C, Osborne CK, Rimawi MF (2020). Towards personalized treatment for early stage HER2-positive breast cancer. Nat Rev Clin Oncol.

[15] Yuan M, Huang LL, Chen JH, Wu J, Xu Q (2019). The emerging treatment landscape of targeted therapy in non-small-cell lung cancer. Signal Transduct Target Ther.

[16] Kayser S, Krzykalla J, Elliott MA, Norsworthy K, Gonzales P, Hills RK (2017). Characteristics and outcome of patients with therapy-related acute promyelocytic leukemia front-line treated with or without arsenic trioxide. Leukemia.

[17] Okines A, Cunningham D, Chau I (2011). Targeting the human EGFR family in esophagogastric cancer. Nat Rev Clin Oncol.

[18] Ilson DH, Kelsen D, Shah M, Schwartz G, Levine DA, Boyd J (2011). A phase 2 trial of erlotinib in patients with previously treated squamous cell and adenocarcinoma of the esophagus. Cancer.

[19] Dutton SJ, Ferry DR, Blazeby JM, Abbas H, Dahle-Smith A, Mansoor W (2014). Gefitinib for oesophageal cancer progressing after chemotherapy (COG): a phase 3, multicentre, double-blind, placebo-controlled randomised trial. Lancet Oncol.

[20] Wells A, Kopp N, Xu X, O’Brien DR, Yang W, Nehorai A (2015). The anatomical distribution of genetic associations. Nucleic Acids Res.

[21] Dobin A, Davis CA, Schlesinger F, Drenkow J, Zaleski C, Jha S (2013). STAR: ultrafast universal RNA-seq aligner. Bioinformatics.

[22] Li H, Handsaker B, Wysoker A, Fennell T, Ruan J, Homer N (2009). The sequence alignment/map format and SAMtools. Bioinformatics.

[23] Anders S, Pyl PT, Huber W (2015). HTSeq-A python framework to work with high-throughput sequencing data. Bioinformatics.

[24] Love MI, Huber W, Anders S (2014). Moderated estimation of fold change and dispersion for RNA-seq data with DESeq2. Genome Biol.

[25] Subramanian A, Tamayo P, Mootha VK, Mukherjee S, Ebert BL, Gillette MA (2005). Gene set enrichment analysis: a knowledge-based approach for interpreting genome-wide expression profiles. Proc Natl Acad Sci USA.

[26] Chen EY, Tan CM, Kou Y, Duan Q, Wang Z, Meirelles GV (2013). Enrichr: interactive and collaborative HTML5 gene list enrichment analysis tool. BMC Bioinformatics.

[27] Najafova Z, Tirado-Magallanes R, Subramaniam M, Hossan T, Schmidt G, Nagarajan S (2017). BRD4 localization to lineage-specific enhancers is associated with a distinct transcription factor repertoire. Nucleic Acids Res.

[28] Hamdan FH, Johnsen SA (2018). DeltaNp63-dependent super enhancers define molecular identity in pancreatic cancer by an interconnected transcription factor network. Proc Natl Acad Sci USA.

[29] Langmead B, Trapnell C, Pop M, Salzberg SL (2009). Ultrafast and memory-efficient alignment of short DNA sequences to the human genome. Genome Biol.

[30] Ramírez F, Dündar F, Diehl S, Grüning BA, Manke T. DeepTools: a flexible platform for exploring deep-sequencing data. Nucleic Acids Res. 2014;42:W187–W191. 10.1093/nar/gku365 PMC4086134 24799436

[31] Zhang Y, Liu T, Meyer CA, Eeckhoute J, Johnson DS, Bernstein BE, et al. Model-based analysis of ChIP-Seq (MACS). *Genome Biol*. 2008;9:R137. 10.1186/gb-2008-9-9-r137 PMC2592715 18798982

[32] Heinz S, Benner C, Spann N, Bertolino E, Lin YC, Laslo P (2010). Simple combinations of lineage-determining transcription factors prime cis-regulatory elements required for macrophage and B cell identities. Mol Cell.

[33] Whyte WA, Orlando DA, Hnisz D, Abraham BJ, Lin CY, Kagey MH (2013). Master transcription factors and mediator establish super-enhancers at key cell identity genes. Cell.

[34] Ernst J, Kellis M (2012). ChromHMM: automating chromatin-state discovery and characterization. Nat Methods.

[35] Mumbach MR, Rubin AJ, Flynn RA, Dai C, Khavari PA, Greenleaf WJ (2016). HiChIP: efficient and sensitive analysis of protein-directed genome architecture. Nat Methods.

[36] Servant N, Varoquaux N, Lajoie BR, Viara E, Chen CJ, Vert JP, et al. HiC-Pro: an optimized and flexible pipeline for Hi-C data processing. Genome Biol. 2015;16:259. 10.1186/s13059-015-0831-x PMC4665391 26619908

[37] Bhattacharyya S, Chandra V, Vijayanand P, Ay F Identification of significant chromatin contacts from HiChIP data by FitHiChIP. Nat Commun. 2019;10:4221. 10.1038/s41467-019-11950-y PMC6748947 31530818

[38] Pivot-Pajot C, Caron C, Govin J, Vion A, Rousseaux S, Khochbin S (2003). Acetylation-dependent chromatin reorganization by BRDT, a testis-specific bromodomain-containing protein. Mol Cell Biol.

[39] Miller TCR, Simon B, Rybin V, Grötsch H, Curtet S, Khochbin S (2016). A bromodomain-DNA interaction facilitates acetylation-dependent bivalent nucleosome recognition by the BET protein BRDT. Nat Commun.

[40] Matzuk MM, McKeown MR, Filippakopoulos P, Li Q, Ma L, Agno JE (2012). Small-molecule inhibition of BRDT for male contraception. Cell.

[41] Gaucher J, Boussouar F, Montellier E, Curtet S, Buchou T, Bertrand S (2012). Bromodomain-dependent stage-specific male genome programming by Brdt. EMBO J.

[42] Shang E, Nickerson HD, Wen D, Wang X, Wolgemuth DJ (2007). The first bromodomain of Brdt, a testis-specific member of the BET sub-family of double-bromodomain-containing proteins, is essential for male germ cell differentiation. Development.

[43] Kim J, Bowlby R, Mungall AJ, Robertson AG, Odze RD, Cherniack AD (2017). Integrated genomic characterization of oesophageal carcinoma. Nature.

[44] Barretina J, Caponigro G, Stransky N, Venkatesan K, Margolin AA, Kim S (2012). The Cancer Cell Line Encyclopedia enables predictive modelling of anticancer drug sensitivity. Nature.

[45] Gilkes DM, Semenza GL, Wirtz D (2014). Hypoxia and the extracellular matrix: drivers of tumour metastasis. Nat Rev Cancer.

[46] Hynes RO (2014). Stretching the boundaries of extracellular matrix research. Nat Rev Mol Cell Biol.

[47] Somerville TDD, Xu Y, Miyabayashi K, Tiriac H, Cleary CR, Maia-Silva D (2018). TP63-mediated enhancer reprogramming drives the squamous subtype of pancreatic ductal adenocarcinoma. Cell Rep.

[48] Moses MA, George AL, Sakakibara N, Mahmood K, Ponnamperuma RM, King KE, et al. Molecular mechanisms of p63-mediated squamous cancer pathogenesis. Int J Mol Sci. 2019;20:3590. 10.3390/ijms20143590 PMC6678256 31340447

[49] Filippakopoulos P, Picaud S, Mangos M, Keates T, Lambert JP, Barsyte-Lovejoy D (2012). Histone recognition and large-scale structural analysis of the human bromodomain family. Cell.

[50] Morinière J, Rousseaux S, Steuerwald U, Soler-López M, Curtet S, Vitte AL (2009). Cooperative binding of two acetylation marks on a histone tail by a single bromodomain. Nature.

[51] Matsui S, Utani A, Takahashi K, Mukoyama Y, Miyachi Y, Matsuyoshi N (2008). Knockdown of Fat2 by siRNA inhibits the migration of human squamous carcinoma cells. J Dermatol Sci.

[52] Hnisz D, Abraham B, Lee T, Lau A, Saint-Andre V, Sigova A (2014). Transcriptional super-enhancers connected to cell identity and disease. Cell.

[53] Jiang Y, Jiang YY, Xie JJ, Mayakonda A, Hazawa M, Chen L, et al. Co-activation of super-enhancer-driven CCAT1 by TP63 and SOX2 promotes squamous cancer progression. Nat Commun. 2018;9:3619. 10.1038/s41467-018-06081-9 PMC6127298 30190462

[54] Schmitt AD, Hu M, Jung I, Xu Z, Qiu Y, Tan CL (2016). A compendium of chromatin contact maps reveals spatially active regions in the human genome. Cell Rep.

[55] Beagrie RA, Scialdone A, Schueler M, Kraemer DCA, Chotalia M, Xie SQ (2017). Complex multi-enhancer contacts captured by genome architecture mapping. Nature.

[56] Winter GE, Buckley DL, Paulk J, Roberts JM, Souza A, Dhe-Paganon S (2015). Drug development. Phthalimide conjugation as a strategy for in vivo target protein degradation. Science.

[57] Zengerle M, Chan K-HH, Ciulli A (2015). Selective small molecule induced degradation of the BET bromodomain protein BRD4. ACS Chem Biol.

[58] Scanlan MJ, Altorki NK, Gure AO, Williamson B, Jungbluth A, Chen YT (2000). Expression of cancer-testis antigens in lung cancer: Definition of bromodomain testis-specific gene (BRDT) as a new CT gene, CT9. Cancer Lett.

[59] Chen L, Cai S, Wang JM, Huai YY, Lu PH, Chu Q. BRDT promotes ovarian cancer cell growth. Cell Death Dis. 2020;11:1021. 10.1038/s41419-020-03225-y PMC7705741 33257688

[60] Wan P, Chen Z, Zhong W, Jiang H, Huang Z, Peng D (2020). BRDT is a novel regulator of eIF4EBP1 in renal cell carcinoma. Oncol Rep.

[61] Kanno T, Kanno Y, LeRoy G, Campos E, Sun H-W, Brooks SR (2014). BRD4 assists elongation of both coding and enhancer RNAs by interacting with acetylated histones. Nat Struct Mol Biol.

[62] Aird F, Kandela I, Mantis C, Iorns E, Denis A, Williams SR, et al. Replication study: BET bromodomain inhibition as a therapeutic strategy to target c-Myc. Elife. 2017;6:e21253. 10.7554/eLife.21253 PMC5245966 28100400

[63] Cheng Z, Gong Y, Ma Y, Lu K, Lu X, Pierce LA (2013). Inhibition of BET bromodomain targets genetically diverse glioblastoma. Clin Cancer Res.

[64] Segura MF, Fontanals-Cirera B, Gaziel-Sovran A, Guijarro MV, Hanniford D, Zhang G (2013). BRD4 sustains melanoma proliferation and represents a new target for epigenetic therapy. Cancer Res.

[65] Filippakopoulos P, Qi J, Picaud S, Shen Y, Smith WB, Fedorov O (2010). Selective inhibition of BET bromodomains. Nature.

[66] Piha-Paul SA, Hann CL, French CA, Cousin S, Braña I, Cassier PA, et al. Phase 1 study of molibresib (GSK525762), a bromodomain and extra terminal domain protein inhibitor, in NUT carcinoma and other solid tumors. JNCI Cancer Spectr. 2020;4:pkz093. 10.1093/jncics/pkz093 PMC7165800 32328561

[67] Bolden JE, Tasdemir N, Dow LE, van Es JH, Wilkinson JE, Zhao Z (2014). Inducible in vivo silencing of brd4 identifies potential toxicities of sustained BET protein inhibition. Cell Rep.

[68] Faivre EJ, McDaniel KF, Albert DH, Mantena SR, Plotnik JP, Wilcox D (2020). Selective inhibition of the BD2 bromodomain of BET proteins in prostate cancer. Nature.

[69] Gilan O, Rioja I, Knezevic K, Bell MJ, Yeung MM, Harker NR (2020). Selective targeting of BD1 and BD2 of the BET proteins in cancer and immunoinflammation. Science.

[70] Raina K, Lu J, Qian Y, Altieri M, Gordon D, Rossi AMK (2016). PROTAC-induced BET protein degradation as a therapy for castration-resistant prostate cancer. Proc Natl Acad Sci USA.

[71] Bai L, Zhou B, Yang C-Y, Ji J, McEachern D, Przybranowski S (2017). Targeted degradation of BET proteins in triple-negative breast cancer. Cancer Res.

[72] Han G, Kortylewicz ZP, Enke T, Baranowska-Kortylewicz J (2014). Co-targeting androgen receptor and DNA for imaging and molecular radiotherapy of prostate cancer: in vitro studies. Prostate.

[73] Vultos F, Fernandes C, Mendes F, Marques F, Correia JDG, Santos I (2017). A multifunctional radiotheranostic agent for dual targeting of breast cancer cells. ChemMedChem.

