# Supplementary material for: Bromodomain protein BRDT directs ΔNp63 function and super-enhancer activity in a subset of esophageal squamous cell carcinomas
Source: Cell Death Differ. 2021 Mar 3;28(7):2207–20. doi: 10.1038/s41418-021-00751-w (PMC8257622; doi:10.1038/s41418-021-00751-w)
Supplement: Supplementary file 1 — Supplementary Information [file 41418_2021_751_MOESM1_ESM.docx]

**Bromodomain protein BRDT directs ΔNp63 function and super enhancer activity in a subset of esophageal squamous cell carcinomas**

Xin Wang^1^, Ana P. Kutschat^1^, Moyuru Yamada^2^, Evangelos Prokakis^1^, Patricia Böttcher^1^, Koji Tanaka^2^, Yuichiro Doki^2^, Feda H. Hamdan^3^, Steven A. Johnsen^1,3,^*

^1^Clinic for General, Visceral and Pediatric Surgery, University Medical Center Göttingen, Göttingen, 37077, Germany

^2^Department of Gastroenterological Surgery, Graduate School of Medicine, Osaka University, Osaka, 565-0871, Japan

^3^Gene Regulatory Mechanisms and Molecular Epigenetics Lab, Division of Gastroenterology and Hepatology, Mayo Clinic, Rochester, MN, 55905, USA

**Supplementary materials and methods**

**Cell culture, knock-down, knock-out and over-expression**

KYSE70, KYSE150 and KYSE180 cells were provided by Jessica Eggert (University Medical Center Göttingen, Germany). TE6 cells were provided by Dr. Koji Tanaka (Osaka University, Japan). Cells were cultured in a humidified incubator supplied with 5% CO_2_ at 37°C. RPMI-1640 (Invitrogen) with 10% FBS (Sigma) and 1% penicillin/streptomycin (Sigma) was used to culture KYSE70, KYSE180 and TE6 cells. RPMI/F12 medium (Invitrogen) with 5% FBS (Sigma) and 1% penicillin/streptomycin (Sigma) was used to culture KYSE150 cells. siRNA-mediated knock-down was performed using Lipofectamine RNAiMAX (Invitrogen) and following manufacturer’s instructions. The sequence information of siGENOME siRNAs (Dharmacon) employed is provided in supplementary table S3. CRISPR/Cas9 mediated knock-out was carried out as previously described (1) and the gRNA information is provided in supplementary table S2. The over-expression experiments for RNA-seq were performed by electroporation as previously described (1). The simultaneous over-expression of BRDT and knock-down of ΔNp63 was carried out using Lipofectamine 3000 (Invitrogen) according to manufacturer’s instructions. The control vector pCDNA5/TO was a gift from Prof. Matthias Dobbelstein (University Medical Center Göttingen, Germany), the BRDT over-expression plasmid was a gift from Kyle Miller (Addgene plasmid # 65381; http://n2t.net/addgene:65381 ; RRID:Addgene_65381) (2) and the deltaNp63alpha-FLAG was a gift from David Sidransky (Addgene plasmid # 26979 ; http://n2t.net/addgene:26979 ; RRID:Addgene_26979) (3).

**Proliferation assay**

1,000 cells were seeded in 96-well plates (Corning) and incubated for 5 to 7 days. Cells were imaged and their confluence assessed every 24 hours by Celigo (Brooks Life Sciences System, USA). Each measuring point was normalized to the corresponding confluence measurement at day 0.

**RNA isolation and quantitative real-time PCR (qPCR)**

Patient materials were homogenized using Tissue Lyser (Qiagen). QIAzol was added to cells or lysed tissue, which once lysed were transferred to a new tube. Chloroform (1/5 volume of QIAzol reagent) was added to the tube and the mixture was vortexed thoroughly. After centrifugation, the aqueous phase was transferred to a new tube and an equal volume of isopropanol was added to precipitate RNA. The RNA pellet was washed with 70% ethanol in DEPC water and resuspended in DEPC water. The concentration of RNA was measured using Nanodrop (Denovox). 1µg RNA was used for reverse transcription with M-MuLV reverse transcriptase (NEB) and 1.5 μM 9-mer random primers. For reverse transcription of patient RNA, Reverse Transcription System (Promega) was used. The complementary DNA (cDNA) was then subjected to the following PCR program: 95°C for 2 minutes, 40 cycles of 10 seconds at 95 °C followed by 30 seconds at 60 °C to determine gene expression. *GAPDH* and *ACTB* were used for normalizing qPCR results in Göttingen and Osaka, respectively. The melting curve was determined by reading plates every 0.5 °C from 60 °C to 95 °C. Primers for qPCR experiments were designed with NCBI Primer BLAST (4). The primer sequences are listed in supplementary table S4.

**Protein isolation and western blot**

Protein was isolated by adding RIPA buffer (1% NP40, 0.1% SDS, 0.5 sodium deoxycholate in 1 x PBS) containing protease inhibitors (100 µM N-Ethylmaleimeide, 100 µM Pefabloc, 100 µM β-glycerophosphate and 1 µM Aprotinin/Leupeptin). The protein samples were subjected to sonication (Bioruptor Pico, Diagenode) at high frequency for 10 minutes (30 seconds on and 30 seconds off). The protein was mixed with 6x Lämmli buffer (350 mM Tris-HCl, 30% glycerol, 10% SDS, 9.3% DTT and 0.02% bromophenol blue) and the mixture was heated at 95°C for 5 minutes. The protein samples were then loaded to a polyacrylamide gel and run for separation. After transferring the protein to nitrocellulose membranes (GE Healthcare), the membrane was blocked with 5% milk in 1x TBST (20 mM Tris, 14 mM NaCl, 0.1% Tween 20) prior to overnight incubation with primary antibodes listed in supplemental information. The respective secondary antibody was incubated with the membrane for 1 hour prior to imaging using BioRad gel doc (Biorad).

**Supplemental tables**

**Table S1 Variance of expression and tissue specificity index of epigenetic factors.**

This table is provided as a separate excel file.

**Table S2 Expression of BRDT in ESCC.**

This table is provided as a separate excel file.

**Table S3.** siRNAs used in this study.

| Name | Sequence (5’ to 3’) |
| --- | --- |
| Non-targeting siRNA #5 | UGGUUUACAUGUCGACUAA |
| BRDT #2 | CAAAUCAACUUCAGUAUCU |
| BRDT #3 | GAAAAUGGAUAACCAAGAA |
| BRDT #4 | GUAGAGAGAACACUAAUGA |
| BRDT #18 | GAGAUAAACUUGGGCGAGU |
| TP63 #5 | CAUCAUGUCUGGACUAUUU |
| TP63 #8 | CGACAGUCUUGUACAAUUU |
| FAT2 | Smart pool, Cat. NO: M-011270-00-0005 |

**Table S4.** Primers used in this study (5’ – 3’).

| Name | Forward | Reverse | Purposes |
| --- | --- | --- | --- |
| GPADH | ATGGGGAAGGTGAAGGTCG | GGGGTCATTGATGGCAACAATA | Gene expression |
| BRDT | GAGTCTGAAAGTAGCAGCAGTGA | TATCCTATCTGTGTGACGCCTGT | Gene expression |
| FAT2 | GCCACACAGGTCCACATCTT | GATCTATGGCCTGGACTCGC | Gene expression |
| KRT14 | CAGAGATGTGACCTCCTCCA | CTCAGTTCTTGGTGCGAAGG | Gene expression |
| PTHLH | AGCCGCCGCCTCAAAAG | AGCTGTGTGGATTTCTGCGA | Gene expression |
| TP63 | TTTAGTGAGCCACAGTACACGAA | GAGAGCATCGAAGGTGGAGC | Gene expression |
| hnFAT2 | CCCACCCCCAGTACCTGTAT | AGGACGATGACAGTGGCTTG | Gene expression |
| hnKRT14 | CCATTCACCCACCTTGTTCCT | CCGACCTGGAAGTGAAGATCC | Gene expression |
| hnPTHLH | GATGGGGCACTTACAGGCG | GAGACTGGTTCAGCAGTGGAG | Gene expression |
| BRDT-Ex5 (Osaka) | ATCACCCAGCGCAACAGAAA | CCTTTTGTAACTTGGGCCGC | Gene expression |
| ACTB  (Osaka) | CGCTCTCTGCTCCTCCTGTTC | ATCCGTTGACTCCGACCTTCAC | Gene expression |
| BRDT | CATTCTGTGAGAACAGGGCA | ACCCAAACAGTACAAATTCTACCTA | Genotyping |
| BRDT-gRNA-1 | ATTCTGGCTCATACTTTTCC | NA | Genome editing |
| BRDT-gRNA-2 | TTTATGAATAGACACCTAAG | NA | Genome editing |

**Table S5.** Antibodies used in this study.

| Target | Purpose | Quantity | Source | Catalog |
| --- | --- | --- | --- | --- |
| BRDT | WB, ChIP | 1:1000, 10 μL | Cell signaling | #65133 |
| BRDT | IP | 6 μg | Santa Cruz | sc-515674 |
| IgG | IP | 6 μg | Diagenode | C15410206 |
| p63 | WB, ChIP | 1:1000, 2 μg | Santa Cruz | sc-8431 |
| H3K27ac | ChIP | 1 μg | Diagenode | C15410196 |
| H3K9ac | ChIP | 1 μg | Diagenode | C15410004 |
| H4K5ac | ChIP | 1 μg | Diagenode | C15410025 |
| H3K4me1 | ChIP | 1 μg | Diagenode | C15410194 |
| H3K4me3 | ChIP | 1 μg | Diagenode | C15410003 |
| H3K27me3 | ChIP | 1 μg | Diagenode | C15410069 |
| BRD2 | WB | 1:1000 | Cell signaling | #5848 |
| BRD3 | WB | 1:1000 | Bethyl | A302-368 |
| BRD4 | WB | 1:1000 | Diagenode | C15410337 |
| GAPDH | WB | 1:5000 | Origene | TA802519 |
| KRT14 | WB | 1:50000 | Biolengend | 905301 |
| FLAG | WB | 1:1000 | Sigma | F1804 |
| GFP | WB | 1:1000 | Invitrogen | CAB4211 |

**Supplemental figures**

**
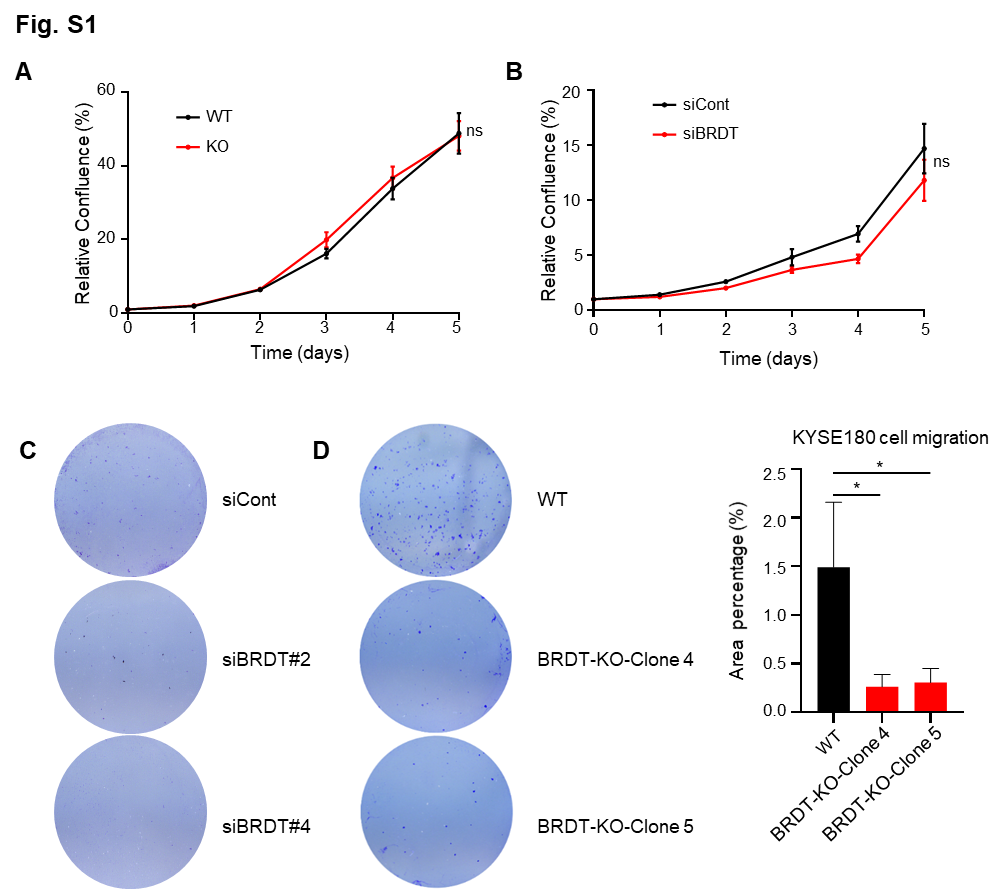
**


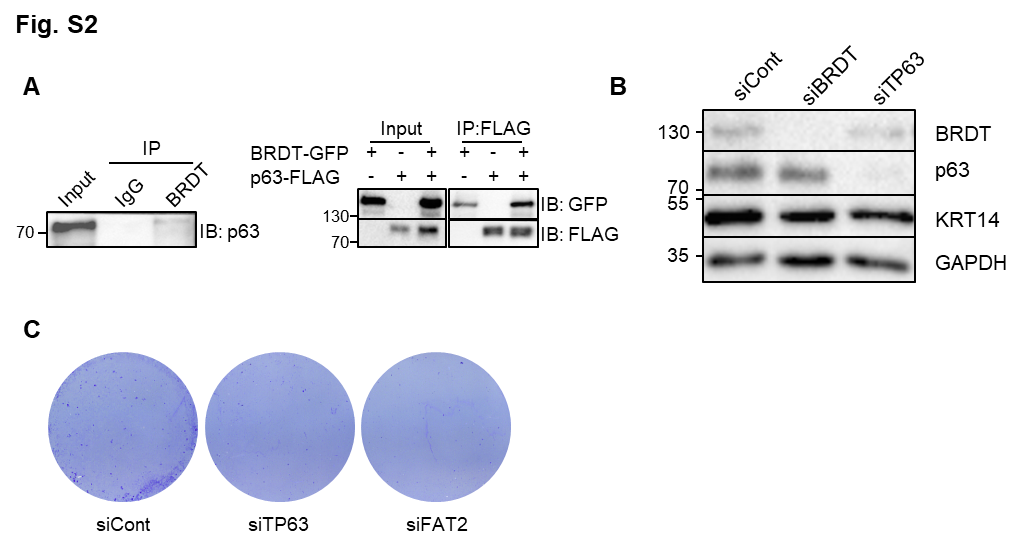


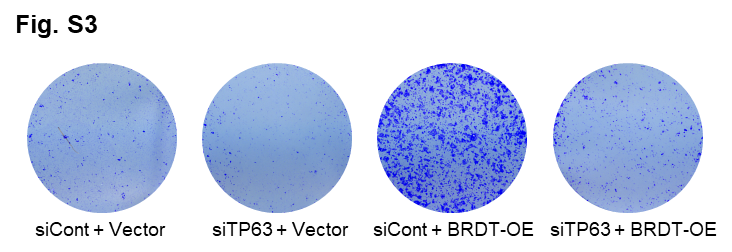


**References**

1. Sen M, Wang X, Hamdan FH, Rapp J, Eggert J, Kosinsky RL, et al. ARID1A facilitates KRAS signaling-regulated enhancer activity in an AP1-dependent manner in colorectal cancer cells. *Clin Epigenetics*. 2019;11:92.

2. Gong F, Chiu L-Y, Cox B, Aymard F, Clouaire T, Leung JW, et al. Screen identifies bromodomain protein ZMYND8 in chromatin recognition of transcription-associated DNA damage that promotes homologous recombination. *Genes Dev*. 2015;29:197–211.

3. Chatterjee A, Upadhyay S, Chang X, Nagpal JK, Trink B, Sidransky D. U-box-type ubiquitin E4 ligase, UFD2a attenuates cisplatin mediated degradation of ΔNp63α. *Cell Cycle*. 2008;7:1231–7.

4. Ye J, Coulouris G, Zaretskaya I, Cutcutache I, Rozen S, Madden TL. Primer-BLAST: a tool to design target-specific primers for polymerase chain reaction. *BMC Bioinformatics*. 2012;13:134.

**Supplemental figure legends**

**Fig. S1** Growth kinetics analysis of CRISPR/Cas9-mediated knock-out of BRDT in KYSE180 (A) and siRNA-mediated knock-down of BRDT in TE6 (B). Data are represented as mean ± SD, n=5. Paired t-test was used. ****: *P*≤0.0001, ***: *P*≤0.005, **: *P*≤0.01, *: *P*≤0.05, ns: not significant. (C) Representative images of migrated cells upon BRDT knock-down with different siRNAs in KYSE180. (D) Representative images of migrated cells from two BRDT knock-out clones and the respective quantification. Data are represented as mean ± SD, n=2. Unpaired one-way ANOVA test followed by Dunnett’s test was used. ****: P≤0.0001, ***: P≤0.005, **: P≤0.01, *: P≤0.05, ns: not significant.

**Fig. S2** (A) Endogenous CoIP (Left) in KYSE180 and exogenous CoIP (Right) in HEK293T showing that BRDT is associated with p63. (B) Western blot analysis of BRDT, p63, KRT14 and GAPDH upon knock-down of p63 or BRDT in KYSE180. (C) Representative images of migrated cells upon knock-down of p63 or FAT2 in KYSE180.

**Fig. S3** Representative images of migrated cells upon over-expression of BRDT and/or knock-down of p63 in KYSE150.
